# A Highly Sensitive Triboelectric Quasi‐Zero Stiffness Vibration Sensor with Ultrawide Frequency Response

**DOI:** 10.1002/advs.202301199

**Published:** 2023-05-03

**Authors:** Pengfan Wu, Fayang Wang, Shiwei Xu, Tao Liu, Youchao Qi, Xue Zhao, Chi Zhang, Xiaojing Mu

**Affiliations:** ^1^ Key Laboratory of Optoelectronic Technology & Systems Ministry of Education International R&D Center of Micro–Nano Systems and New Materials Technology Chongqing University Chongqing 400044 China; ^2^ CAS Center for Excellence in Nanoscience Beijing Key Laboratory of Micro–Nano Energy and Sensor Beijing Institute of Nanoenergy and Nanosystems Chinese Academy of Sciences Beijing 101400 China; ^3^ School of Mechanical and Power Engineering Chongqing University of Science and Technology Chongqing 401331 China

**Keywords:** quasi‐zero stiffness, self‐powered system, triboelectric nanogenerator, vibration sensor

## Abstract

Sensors based on triboelectric nanogenerators (TENGs) have gained worldwide interest owing to their advantages of low cost and self‐powering. However, the detection of most triboelectric vibration sensors (TVS) is restricted to low frequency, whereas high‐frequency vibration signals are successfully measured in recent studies; their sensitivity still requires improvement. Hence, a highly sensitive vibration sensor based on TENG (HSVS‐TENG) with ultrawide frequency response is presented. This study is the first to introduce a quasi‐zero stiffness structure into the TENG to minimize the driving force by optimizing the magnetic induction intensity and the weight of the moving part. The results show that the HSVS‐TENG can measure vibrations with frequencies ranging from 2.5 to 4000 Hz, with a sensitivity ranging from 0.32 to 134.9 V g^−1^. Moreover, the sensor exhibits a good linear response versus the applied acceleration, and the linearity ranges from 0.08 to 2.81 V g^−1^. The self‐powered sensor can monitor the running state and fault type of the key components with a recognition accuracy of 98.9% by leveraging machine‐learning algorithms. The results reach a new height for the ultrawide frequency response and high sensitivity of the TVS and provide an idea for a follow‐up high‐resolution TVS.

## Graphical abstract

A highly sensitive triboelectric quasi‐zero stiffness vibration sensor is presented. By the design, it reaches a new height for the ultrawide frequency response (≈4000 Hz) and high sensitivity (≈134.9 V g^−1^). The sensor can monitor the running state and fault type of the key components with a recognition accuracy of 98.9% by leveraging machine‐learning algorithms.

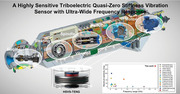

## 1 Introduction

Vibration, a common phenomenon in the universe, can provide much information about its source. Vibration sensors convert vibration signals into electrical signals, which have been widely applied in the health condition monitoring fields of marine environments,^[^ ^1^ ^]^ mechanical equipment,^[^ ^2^ ^]^ large civil structures,^[^ ^3^ ^]^ and transporting vehicles.^[^ ^4^ ^]^ However, with the booming development of the Internet of Things,^[^ ^5^, ^6^, ^7^ ^]^ traditional sensors struggle to meet trillions of sensor network requirements owing to maintenance difficulties, high costs, additional energy supplies, and limited lifetime.^[^ ^8^, ^9^, ^10^ ^]^ Hence, it is highly desirable to develop a low‐cost, self‐powered vibration sensor that does not require maintenance or an additional energy supply.

Recently, triboelectric nanogenerators (TENGs) have proven to be a simple configuration, cost‐effective fabrication, and lightweight way to detect vibration.^[^ ^11^, ^12^, ^13^ ^]^ In addition, most ambient triboelectric vibration sensors (TVS) are limited to a low‐frequency range (below several hundred hertz).^[^ ^14^, ^15^, ^16^, ^17^ ^]^ In the low‐frequency band, these sensors exhibit much higher sensitivity than vibration sensors based on other principles such as electromagnetic induction, capacitive, and optical fiber.^[^ ^18^, ^19^ ^]^ To broaden the measuring vibration frequency range of TENG‐based vibration sensors, researchers have been committed to designing multiple degrees of freedom,^[^ ^20^, ^21^ ^]^ ball bouncing,^[^ ^22^, ^23^ ^]^ resonant structures,^[^ ^24^, ^25^ ^]^ and flexible film.^[^ ^26^, ^27^, ^28^ ^]^ The most recent state‐of‐the‐art TVS reached 2 kHz frequency signal output, but its sensitivity (≈0.01 V g^−1^ at more than a few kilohertz of vibration frequencies) still required improvement.^[^ ^44^ ^]^ Regardless of the structure, getting a wider bandwidth requires a smaller driving resistance force, and obtaining greater sensitivity requires sufficient mass to obtain energy. Therefore, the balance between a small driving resistance force and sufficient mass remains a challenge, which is a key question in designing a self‐powered sensor capable of measuring a wider frequency range and higher sensitivity to expand their application.

A highly sensitive vibration sensor based on a TENG (HSVS‐TENG) with an ultrawide bandwidth was presented in this study. We introduce a quasi‐zero stiffness structure to minimize the driving force required for the contact–separation TENG by optimizing the magnet strength and the weight of the stainless steel, and the repulsive force of the magnet is equal to the dead weight when the gap is zero. At this time, the initial equivalent stiffness of the system is close to zero. The results show that the HSVS‐TENG can measure vibrations with frequencies ranging from 2.5 to 4000 Hz with the acceleration as low as 0.1 g, and the output signal shows good linearity at different accelerations. With a recognition accuracy of 98.9%, it has completed an exploratory application in detecting the running states and fault types of key components (including the motor, reducer, bearing, compressor, and blast engine) using a machine‐learning algorithm. The sensor network composed of the HSVS‐TENG modules is expected to realize the potential application of control–logic feedback and fault monitoring by detecting the key components of the major equipment. This work not only reaches a new height of the wide‐frequency response and high sensitivity of the TVS but also provides an idea for a follow‐up high‐resolution TVS.

## 2 Result and Discussion

### 2.1 Structure and Working Principle

**Figure 1:**
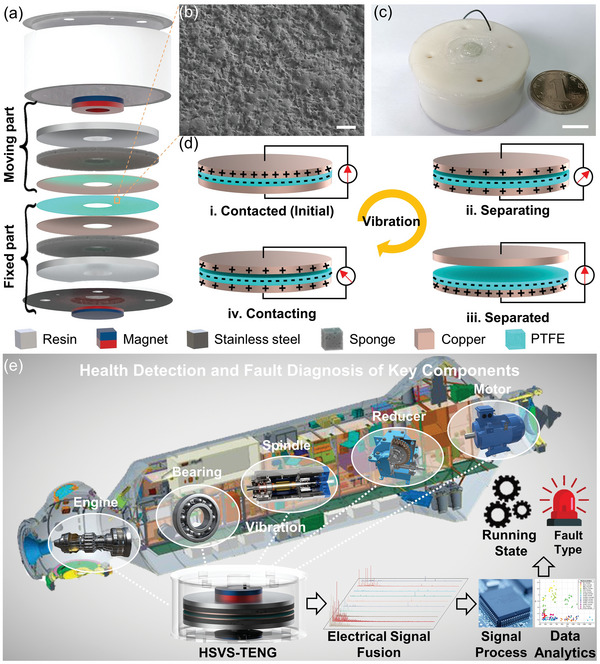
Structure and working mechanism of the HSVS‐TENG. a) Structure diagram of the HSVS‐TENG. b) Scanning electron microscope (SEM) image of the PTFE surface. Scale bar, 20 µm. c) Photograph of the HSVS‐TENG. Scale bar, 1 cm. d(i)–(iv)) Working principle of the TENG. e) Application of the HSVS‐TENG in the fault diagnosis of key components of the major equipment.

A structural diagram and photograph of the HSVS‐TENG are shown in **Figure** **1**a and Figure 1b, respectively. The HSVS‐TENG is cylindrical with a diameter of 40 mm and a height of 20 mm, and its volume is ≈25.12 cm^3^. The working process of the HSVS‐TENG was based on the contact–separation mode. As shown in Figure 1a,b, the moving part and the fixed part are repelled by the two magnets to construct the TENG. The moving part consisted of a levitated magnet, a stainless‐steel gasket, a piece of sponge, and a piece of copper foil. The stainless‐steel gasket was used as a mass. The sponge acted as a buffer to ensure that the two friction materials were in complete contact. The copper foil acted as both the friction material and the electrode, and the levitated magnet was attached to the stainless steel to move the electrode up and down. Furthermore, a fixed magnet, stainless‐steel gasket, a piece of sponge, polytetrafluoroethylene (PTFE) film, and a piece of copper foil form the fixed part. The magnet was fixed at the bottom of the base. In addition, as a tribo‐charge layer, the PTFE surface was prepared for the nanostructure (shown in Figure 1b) using a 10 000 mesh sandpaper to enhance the surface charge density for higher sensitivity. The comprehensive comparison of PTFE properties (surface morphology, open‐circuit voltage, short‐circuit current, and transfer charge and surface charge density) with and without treatment which is shown in Figure S1 (Supporting Information). The shell and base were fabricated using photosensitive resin, manufactured by 3D printing. The parameter optimization of the initial gap between the two friction materials (PTFE and copper) for a wide‐frequency response device is in Section 2.2.

The working principle of the HSVS‐TENG based on the contact and separation modes is illustrated schematically in Figure 1d. In the initial condition, the gap between the two tribo‐charged materials was close to zero, and no charge was generated; thus, there was no potential difference between the two electrodes. When the vibration occurred, the copper foil came to contact with the PTFE film for the second time, initiating the working cycle. Electron clouds on the surfaces of the two films overlapped with each other, and some electrons from cuprum entered the PTFE surface due to its deeper potential well. Owing to the electronegativity of PTFE, which was much larger than that of copper, free electrons on the cuprum surface were transferred to the lowest unoccupied molecular orbital at the PTFE interface so that the PTFE film was negatively charged and the copper was positively charged (Figure 1d(i)). Because the magnetic force on the moving part counteracts gravity as a spring when the external vibration source loads on the device, the levitated magnet starts to move the mass and the electrode up, and cuprum begins to separate from the PTFE film (Figure 1d(ii)). At this moment, electrons on the PTFE film had a potential difference between the upper and lower copper electrodes due to the electrostatic induction; thus, the electron flow was driven by the potential difference. Conversely, when the vibration source moves downward, the displacement of the moving parts reaches the maximum value (Figure 1d(iii)); cuprum begins to close to the PTFE film; and current flows from the top electrode to the bottom electrode because of the potential difference in the local electric field (Figure 1d(iv)). Finally, the moving part was restored to the original position; the charge distribution was restored to the initial state (Figure 1d(i)); and the alternating current power signal generation cycle was completed. According to Gauss's law, the electric potential difference between the two cuprum electrodes can be analytically expressed as^[^ ^29^ ^]^

(1) $$V_{\mathrm{oc}}=\frac{\sigma D(t)}{\varepsilon }$$

where *σ* is the surface charge density of the PTFE film, *D*(*t*) is the gap distance between the two tribo‐materials, and *ε* is the dielectric constant in a vacuum. To further verify the working principle of the HSVS‐TENG, the electric field distribution between the PTFE film and the cuprum foil under different gaps was simulated using COMSOL 6.0 (shown in Figure S2a in the Supporting Information).

A schematic illustration of the health detection and fault diagnosis system for the critical components based on the HSVS‐TENG is shown in Figure 1e. The vibration of different components during the operation was converted into electrical signals by the TVS. Finally, the running states or fault types of each key component were obtained through signal processing and data analysis.

### 2.2 Theory Model and Parameters’ Optimization

**Figure 2:**
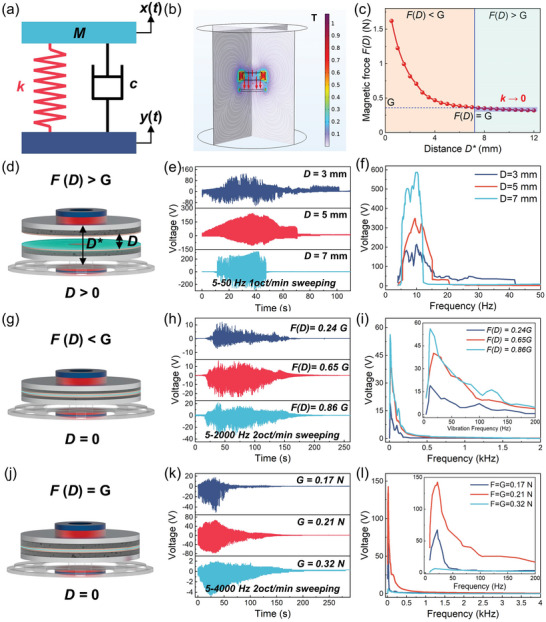
Theory model and parameters optimization. a) Schematic diagram of the dynamic model. b) COMSOL simulation of the magnetic field distribution. c) Simulation result of the repulsive magnetic force as a function of the distance. d) Schematic illustration of the TENG in the force of the magnet is greater than its weight, with the corresponding e) output voltage signal under vibration sweep frequency with the different gap distance and f) voltage signal as a function of vibration frequency with different gap distances. g) Schematic illustration of the TENG when the magnetic force is less than its weight, with the corresponding h) output voltage signal under vibration frequency sweeping with the different magnetic force, and i) voltage signal with the function of vibration frequency. The inset of the figure shows the magnified view of the output signal which less than 200 Hz. j) Schematic illustration of the TENG when the magnetic force is equal to its weight, with the corresponding k) output voltage signal under vibration sweep frequency with different gravity of the moving part, and l) voltage signal with a function of vibration frequency; the inset of the figure shows the magnified view of the output signal which less than 200 Hz.

The HSVS‐TENG was modeled as a single‐degree‐of‐freedom system with nonlinear stiffness to study its frequency response characteristics. The model is represented as a mass–spring–damper system with mass block *M*, spring stiffness *k*, and damping factor *c* (shown in **Figure** **2**a). The gravity of mass block *G* is the sum of the gravity of the components of the moving part. The equivalent spring stiffness *k* is the ratio of the difference between the repulsive force of the magnets *F*(*D*) and the gravitational force *G* to the displacement of the friction material *D*. The dynamic equation of the HSVS‐TENG system can be established as follows

(2) $$M\ddot{x}+c\dot{x}+F\left(D\right)-G=-M\ddot{y}$$

where *x* is the vibration amplitude of the exciting source, and *y* is the displacement of the moving part relative to the fixed part. The repulsive force between the two magnets *F*(*D*) can be expressed as^[^ ^30^ ^]^

(3) $$F\left(D\right)=\frac{\mu Q_{1}Q_{2}}{4\pi D{*}^{2}}$$

where *µ* is the vacuum permeability, and *Q* is the magnetic field intensity of the magnet (Figure 2b; Figure S2, Supporting Information). The relationship between the distance of the repulsive magnets D* and the gap of the tribo‐materials *D* can be expressed as

(4) D∗=D+B

where *B* is the sum of the thicknesses of the weights, sponges, friction material, electrodes, and lower base. The repulsive force of the magnets was simulated and calculated using COMSOL 6.0, as shown in Figure 2c. The equivalent stiffness of the system can be calculated by

(5) $$k=\frac{\partial F(D)}{\partial D}$$

Combined with the electric output characteristics (Equation (1)) and dynamic model (Equation (2)) of the TENG, the output voltage signal of the self‐powered sensor under sinusoidal vibration can be expressed as

(6) $$V_{\mathrm{oc}}=\frac{\sigma A\omega^{2}}{\varepsilon \sqrt{{\left(\frac{k}{M}-\omega^{2}\right)}^{2}+4\frac{c^{2}}{M^{2}}\omega^{2}}}\cos\left(\omega t+\phi \right)$$

According to the characteristics of the sinusoidal vibration and electrodynamic shaker,^[^ ^31^ ^]^ the relationships between the vibration frequency, acceleration, and displacement can be expressed as

(7) $$a=A{\left(2\pi f\right)}^{2}$$

where *A* is the displacement amplitude of the moving table, *f* is the vibration frequency, and *a* is vibration acceleration.

From Equation (7), when the system vibration frequency is higher than 100 Hz, the external vibration excitation amplitude is at the micron level (Figure S4, Supporting Information), and the equivalent stiffness is close to zero when the distance between the two magnets increases to a certain value. At this point, the system approached a critical state that was not resonant. Owing to the quasi‐zero stiffness structure, the system exhibits superior frequency response performance in the middle‐ and high‐frequency bands.

To verify the above dynamic theoretical model, we systematically investigated the output voltage signal and basic characteristics of the HSVS‐TENG under different relationships between the magnetic force *F*(*D*) and gravity of the moving parts *G* (shown in Figure 2c). When the gap between the tribo‐materials was greater than zero, the repulsive magnetic force was greater than gravity (see Figure 2d), and the output voltage signals of the TENG under a sinusoidal vibration sweep frequency ranging from 5 to 50 Hz at a frequency sweep rate of 1 oct min^−1^ (Figure 2e). The corresponding frequency spectra of the TENG with various gap distances between the tribo‐materials from the Fourier transform are shown in Figure 2f. From the typical output voltage of the resonant device, the TENG exhibited a high‐sensitivity performance, but the resonant feature leads to a narrow frequency band (which is less than 50 Hz). In addition, as the gap distance *D* increases, the output voltage signal frequency range decreases, and the resonant frequency output voltage increases. The results showed that the TENG with a large magnetic force had a high voltage signal output and narrow frequency response range. To broaden the vibration frequency width of the self‐powered sensor output voltage signals, the output performance was studied with a gap distance of zero. Figure 2g shows the foam compression deformation because the magnetic force *F*(*D*) is less than its gravity *G* (while the gap distance *D* is zero). The time domain and frequency domain of the output voltage signals by the TENG under sinusoidal frequency sweeping vibration (ranging from 5 to 2000 Hz with a sweeping rate of 2 oct min^−1^) are shown in Figure 2h,i. As the magnetic force increases, the sensor sensitivity and bandwidth increase. To verify the above theoretical model, we further discuss the initial critical state (Figure 2j) in which gravity is equal to magnetic force (*F*(*D*) = *G*) and the separation is zero (*D* = 0). The experimental output results under sinusoidal frequency sweeping vibration are shown in Figure 2k,i. The results showed that optimizing the gravity of the moving part (*G* = 0.21 N) of the self‐powered sensor can improve the sensitivity and frequency bandwidth (reaching 4000 Hz). The quasi‐zero stiffness mechanism can maximize the bandwidth of the device and further optimize the sensitivity in the critical state.

### 2.3 Output Performance of the TENG

To systematically characterize the output performance and response of the HSVS‐TENG, a vibration excitation and measurement experiment platform was built (shown in Figure S3 in the Supporting Information). The HSVS‐TENG and accelerometer were mounted on the shaker, and the frequency and acceleration of the shaker were precisely set by the vibration control system (which forms a closed‐loop system with a signal generator and power amplifier through the accelerometer).

**Figure 3:**
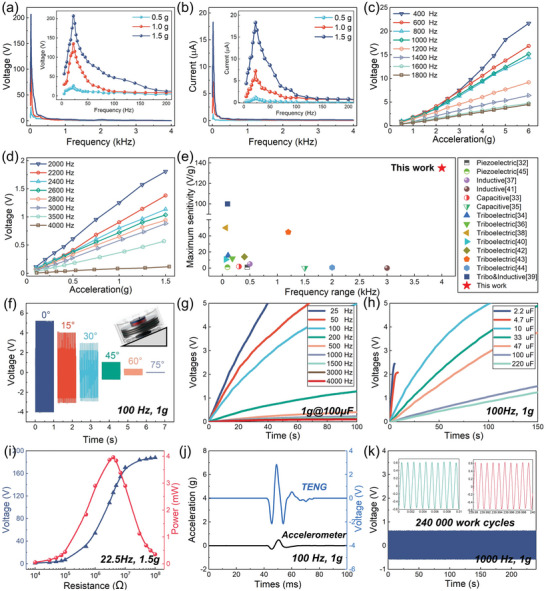
Output performance and characteristics of the HSVS‐TENG. a) Open‐circuit voltage and b) short‐circuit current with a function of vibration frequency under different accelerations. The inset of the figure shows the magnified view of the output signal, which is less than 220 Hz. c,d) Open‐circuit voltage of the HSVS‐TENG with a function of acceleration under different frequencies. e) Comparison of maximum sensitivity and frequency ranges between this work and other vibration sensors. f) Output voltage of the HSVS‐TENG when the angle between the device and vibration source is from 0 to 75°. g) Voltage charge curve of a 10 µF capacitor under different frequencies of vibration with excitation acceleration of 1 g. h) Voltage charge curve of different capacitors under a vibration frequency of 100 Hz with the excitation acceleration of 1 g. i) Peak power and voltage response of the TENG under various resistances. j) Output voltage of the HSVS‐TENG excited by 1 g pulse acceleration. k) Open‐circuit voltage of the HSVS‐TENG over 240 000 working cycles under the high frequency of 1000 Hz.

The output open‐circuit voltage and short‐circuit current of the HSVS‐TENG with vibration frequencies ranging from 2.5 to 4000 Hz under different accelerations were measured, as shown in **Figure** **3**a,b and in Video S1 (Supporting Information). The peak output electrical signals of the HSVS‐TENG at acceleration rates of 0.5, 1, and 1.5 g, show the same trend (which is shown in the magnified views of Figure 3a,b), and the maximum output open‐circuit voltage and short‐circuit current are 207.11 V and 18.23 µA under the vibration frequency of 22.5 Hz, with an acceleration of 1.5 g. Furthermore, Figure 3a,b shows the corresponding curve of frequency obtained through point by point, the time‐domain signals for the main points in Figure 3a are shown in Figures S5–S7 (Supporting Information). To further verify the linearity between the output voltage signal and exciting vibration acceleration of the HSVS‐TENG under different frequencies, we measured the open‐circuit voltage of the HSVS‐TENG with a function of acceleration from 0.5 to 6 g under the vibration frequency range from 400 to 1800 Hz and linearity range from 0.75 to 2.81 V g^−1^ (Figure 3c). In addition, Figure 3d shows the relationship between the output voltage of the HSVS‐TENG and different accelerations ranging from 0.1 to 1.5 g at the vibration frequency ranging from 2000 to 4000 Hz. The output voltage signal has good linearity with various vibration accelerations when the frequency is higher than 400 Hz and the linearity of the HSVS‐TENG ranges from 0.08 to 2.81 V g^−1^. In particular, the HSVS‐TENG could detect a high‐frequency and small‐acceleration vibration signals (the frequency of up to 4000 Hz and acceleration as low as 0.1 g), and the results are shown in Figure 3d, and in Figure S8 and Video S2 (Supporting Information). Significant progress has been made in self‐powered vibration sensors.^[^ ^32^, ^33^, ^34^, ^35^, ^36^, ^37^, ^38^, ^39^, ^40^, ^41^, ^42^, ^43^, ^44^, ^45^ ^]^ Compared with the relevant studies, the HSVS‐TENG shows remarkable performance for measuring a wider frequency range, with lower acceleration rates, and higher sensitivity (shown in Figure 3e and in Table S1 in the Supporting Information).

All the above experiments on the HSVS‐TENG were conducted with vertical vibration, while the output of the self‐powered sensor in other angle vibrations was also measured, as shown in Figure 3f. The results show the HSVS‐TENG can be applied to vibration monitoring scenarios where the vibration angle is less than 60°, which also broadens the application scenarios of the sensor. To verify the availability of the HSVS‐TENG voltage signal, we used a rectifier bridge to connect the TENG to the capacitor, and the voltage charge curve of a 10 µF capacitor under different vibration frequencies with an excitation acceleration of 1 g was measured (Figure 3g). Figure 3h shows the voltage charge curve of different capacitors under a vibration frequency of 100 Hz with an excitation acceleration of 1 g. The power response of the TENG at different impedance values under a resonance frequency with an acceleration rate of 1.5 g is shown in Figure 3i. The peak output power of the TENG reached 3.96 mW with a load resistance of 4 MΩ, and other vibration frequencies are shown in Figure S9 (Supporting Information). The output voltage of the accelerometer (with a charge amplifier gain of 100 mV g^−1^ and a sensitivity of 50 pC g^−1^) was compared with that of the HSVS‐TENG in one pulse acceleration (shown in Figure 3j and in Figure S10a–d in the Supporting Information). To better reflect the advantage of the TENG in ultrawide frequency response, the further comparison results under high‐frequency vibration (1–4 kHz) are shown in Figure S10e–h (Supporting Information). Moreover, a comparison of their response performance under random vibration is also presented in Video S3 (Supporting Information). The results showed that the voltage signal of the HSVS‐TENG exhibited good synchronization and higher sensitivity than that of the accelerometer. A higher output can significantly simplify the signal processing circuit, thereby reducing the detection cost. To study the stability and durability of the device, 24 000 working cycles were conducted under a vibration frequency of 10 Hz and an acceleration rate of 1 g at a monthly interval. Figure 3k shows the open‐circuit voltage of the HSVS‐TENG over 240 000 working cycles under the high frequency of 1000 Hz. The output voltage of the TENG showed no significant change from 1 month ago (shown in Figure S11 in the Supporting Information). The HSVS‐TENG exhibited high durability and good accuracy retention.

### 2.4 Self‐Powered Health Detection and Fault Diagnosis System

**Figure 4:**
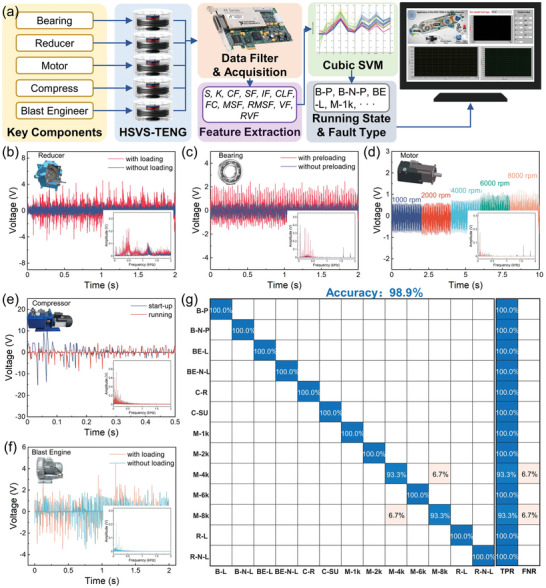
Application of the HSVS‐TENG in running state and fault diagnosis of the key components. a) Work process of the self‐powered health detection and fault diagnosis system with the machine‐learning algorithm. Time‐domain and frequency‐domain signals of the open‐circuit voltage output by the self‐powered sensor mount at b) reducer with/without loading (R‐L/R‐N‐L), c) bearing with/without preloading (B‐P/B‐N‐P), d) motor with different revolution speed (200–2500 rpm, M‐200, M‐500, M‐1k, M‐2k, M‐2.5k), e) compressor with start‐up/running (C‐SU/C‐R), and f) blast engine with/without loading (BE‐L/BE‐N‐L). g) Confusion matrix of the machine‐learning outcome of classing the running states and fault types of the key components.

The work process of the self‐powered health detection and fault diagnosis system using the machine‐learning algorithm is shown in **Figure** **4**a. In this system, the key components of the major equipment generate vibrations owing to the running state switches or running failures. The voltage signals are converted from the vibration of the key components using the HSVS‐TENG. The analog voltage signals are converted to digital signals using the date filter and acquisition. To further extract and identify the information contained in the voltage signal, we extracted the features of the collected signals from the time‐domain and frequency‐domain signals (details are shown in Note S1 in the Supporting Information). The acquired signal has 11 dimensionless features: skewness (*S*), kurtosis (*K*), crest factor (CF), shape factor (SF), impulse factor (IF), clearance factor (CLF), frequency centroid (FC), mean square frequency (MSF), root mean square frequency (RMSF), variance frequency (VF), and root variance frequency (RVF). Then, the signals that represent the information (the running state and fault type) of the vibration source were analyzed and recognized by a machine‐learning algorithm (cubic support vector machine, cubic SVM). The detailed machine‐learning process is shown in Note S2 (Supporting Information).

To achieve fault monitoring of critical components of major equipment, we placed a planetary gear reducer as a vibration source detected by the HSVS‐TENG during weight loading (R‐L) or without loading (R‐N‐L). The time domain and frequency domain of the output voltage are shown in Figure 4b and in Figure S12 (Supporting Information). The heavy load on the output of the harmonic reducer caused the vibration to be more severe in both the time‐ and frequency‐domain signals. Figure 4c and Figure S13 (Supporting Information) show the self‐powered sensor's output signal from the vibration of a deep groove ball bearing with (B‐P) or without preloading (B‐N‐P). The comparison shows that the amplitude of the time‐domain signals after preloading was much larger than that without preloading, and the frequency‐domain signals were concentrated below 500 Hz. The HSVS‐TENG was also used to measure a servo motor with different rotational speeds (M‐1k, M‐2k, M‐4k, M‐6k, and M‐8k) as shown in Figure 4d and in Figure S14 (Supporting Information), and the results showed that the output voltage was higher at higher speeds. To provide feedback to the entire equipment control system, we monitored the operation of a compressor using the triboelectric sensor during its start‐up (C‐SU) or running (C‐R), as shown in Figure 4e. The complete duration of the output signal comparison is shown in Figure S15 (Supporting Information). Figure 4f shows the different vibration self‐powered sensing signal output of a blast engine under load (BE‐L) and no‐load conditions (BE‐N‐L). The entire signal is shown in Figure S16 (Supporting Information). The vibration of the planetary gear reducer and the output of the servo motor voltage signals by the TENG were collected by a digital oscilloscope (Video S4, Supporting Information). Furthermore, the intuitive demonstration for state detection and fault diagnosis by the LabView 2013 is shown in Video S5 (Supporting Information).

After many tests, 11 eigenvalues were extracted from each of the 189 signals output by the HSVS‐TENG to form a feature matrix of 189 × 11. After applying the cubic SVM to the feature matrix, the matrices representing the signals of the key components of the running state or fault type were classified. The machine‐learning result is shown in the confusion matrix (Figure 4g). A high recognition of 98.9% was achieved for identifying the running state or fault type of the key components (13 types of labels). The results indicate that the HSVS‐TENG as a self‐powered sensor can realize the control logic feedback and fault monitoring of major equipment.

## 3 Conclusion

A highly sensitive vibration sensor based on a triboelectric nanogenerator (HSVS‐TENG) with an ultrawide frequency response range was presented in this study. We introduced a quasi‐zero stiffness structure comprising a PTFE film, copper foil, stainless steel, and levitating magnet. By optimizing the magnet strength and weight of the stainless steel, the distance and pressure between the PTFE and copper electrodes were zero in the initial state. Through this structure, the HSVS‐TENG can measure vibrations with frequencies ranging from 2.5 to 4000 Hz, which has a sensitivity ranging from 0.32 to 134.9 V g^−1^. Moreover, The HSVS‐TENG exhibited a good linear response versus the applied acceleration at each of the detecting high frequencies (more than 400 Hz), and the linearity ranged from 0.08 to 2.81 V g^−1^. Furthermore, the TENG output response of single‐cycle vibration pulse vibration signals with different accelerations indicated its highly sensitive responses. The HSVS‐TENG is applied to monitor the running state and fault type of the key components with a recognition accuracy of 98.9%. It indicated that the HSVS‐TENG as a self‐powered sensor has the potential to realize the control logic feedback and fault monitoring of major equipment. The results reach a new height for the ultrawide frequency response and high sensitivity of the TVS (Table S1, Supporting Information) and provide a new idea for a follow‐up high‐resolution TVS.

## 4 Experimental Section

### Numerical Simulations

The structured movement, potential distribution, and repulsive force between the two magnets were numerically calculated using the commercial software COMSOL Multiphysics 6.0. To reduce the number of calculations and improve the calculation efficiency, a 2D axisymmetric model was set for the simulation and the modeled dimensions were consistent with the actual device parameters.

### Fabrication of the HSVS–TENG

All shells and supports were manufactured from the photosensitive resin using 3D printers (Kings JS‐6000‐H). The PTFE film had a thickness of 30 µm and its surface microstructure was hot‐pressed with 10 000 mesh sandpaper to increase its surface potential. Copper foil with a thickness of 50 µm was selected as both the tribomaterial and electrode. The attached sponge (35 mm in diameter and 2 mm in thickness) was the buffer layer between the tribo‐material and base. All parts were assembled, as shown in Figure 1b, to obtain the self‐powered sensor.

### Experimental Process and Measuring Equipment

The surface morphologies of the PTFE film were measured using a field‐emission scanning electron microscope (ZEISS GeminiSEM 300). The sensor was mounted on an electrodynamic shaker (JZK‐40), which was driven by an amplified sinusoidal wave from a signal generator (SINOCERA YE1311) and an amplifier (YE5872A). For the raw data in Figures 2, 3, 4, the output voltage and current signals were measured using a digital oscilloscope (ROHDE& SCHWARZ RTO 2024) and a Keithley 6514 electrometer. The data obtained from the electrometer were collected by a data acquisition card (NI PCI‐6259) on a desktop computer, and LABVIEW 2013 was used to process and display the real‐time signals measured with the electrometer. Feature extraction and cubic SVM machine‐learning algorithms were implemented using MATLAB 2022b. The key components in the application were the harmonic reducer (Harmonic Drive, CSF‐232), deep groove ball bearing (HRB‐6206RZ), direct‐current motor (JJ‐1B 100 W), compressor (Rotary Vane Vacuum Pump 2XZ‐2), and blast engine (YASNIBA HG210‐37AD3).

## Conflict of Interest

The authors declare no conflict of interest.

## Supporting information

Supporting Information

Supplemental Video 1

Supplemental Video 2

Supplemental Video 3

Supplemental Video 4

Supplemental Video 5

## Data Availability

The data that support the findings of this study are available from the corresponding author upon reasonable request.
