# Supplementary material for: A Highly Sensitive Triboelectric Quasi‐Zero Stiffness Vibration Sensor with Ultrawide Frequency Response
Source: Adv Sci (Weinh). 2023 May 3;10(21):2301199. doi: 10.1002/advs.202301199 (PMC10375136; doi:10.1002/advs.202301199)
Supplement: Supplementary file 1 — Supporting Information [file ADVS-10-2301199-s005.pdf]

ADVANCED SCIENCE
Open Access

Supporting Information

for *Adv. Sci.*, DOI 10.1002/advs.202301199

A Highly Sensitive Triboelectric Quasi-Zero Stiffness Vibration Sensor with Ultrawide Frequency Response

Pengfan Wu, Fayang Wang, Shiwei Xu, Tao Liu, Youchao Qi, Xue Zhao, Chi Zhang and Xiaojing Mu**

Supporting Information

A Highly Sensitive Triboelectric Quasi-Zero Stiffness Vibration Sensor with Ultra-Wide Frequency Response

Pengfan Wu[1,2], Fayang Wang[1], Shiwei Xu[1], Tao Liu[1], Youchao Qi[2], Xue Zhao[3], Chi Zhang[2] Xiaojing Mu[1]**

P. Wu[1,2], F. Wang[1], S. Xu[1], T. Liu[1], X. Mu[1*]

[1]Key Laboratory of Optoelectronic Technology & Systems Ministry of Education, International R&D Center of Micro-Nano Systems and New Materials Technology, Chongqing University, Chongqing 400044, China.

Y. Qi[2], C. Zhang[2*]

[2]CAS Center for Excellence in Nanoscience, Beijing Key Laboratory of Micro-nano Energy and Sensor, Beijing Institute of Nanoenergy and Nanosystems, Chinese Academy of Sciences, Beijing 101400, China.

X. Zhao[3]

[3]School of Mechanical and Power Engineering, Chongqing University of Science and Technology, Chongqing 401331, China.

E-mail: mxjacj@cqu.edu.cn; czhang@binn.cas.cn.

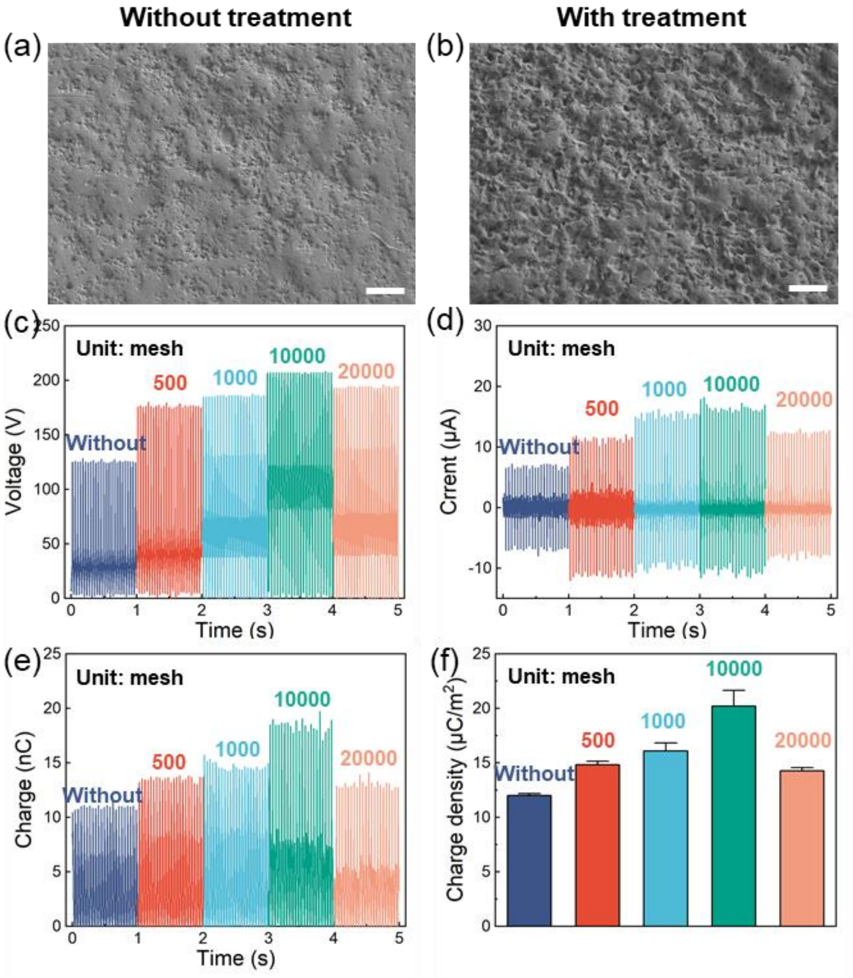

Figure S1 The comprehensive comparison of PTFE properties: (a) open-circuit voltage, (b) short-circuit current, (c) transfer charge and (d) surface charge density) with different treatments. The surface morphology (e) without treatment and with treatment by the 10000 mesh sandpaper.

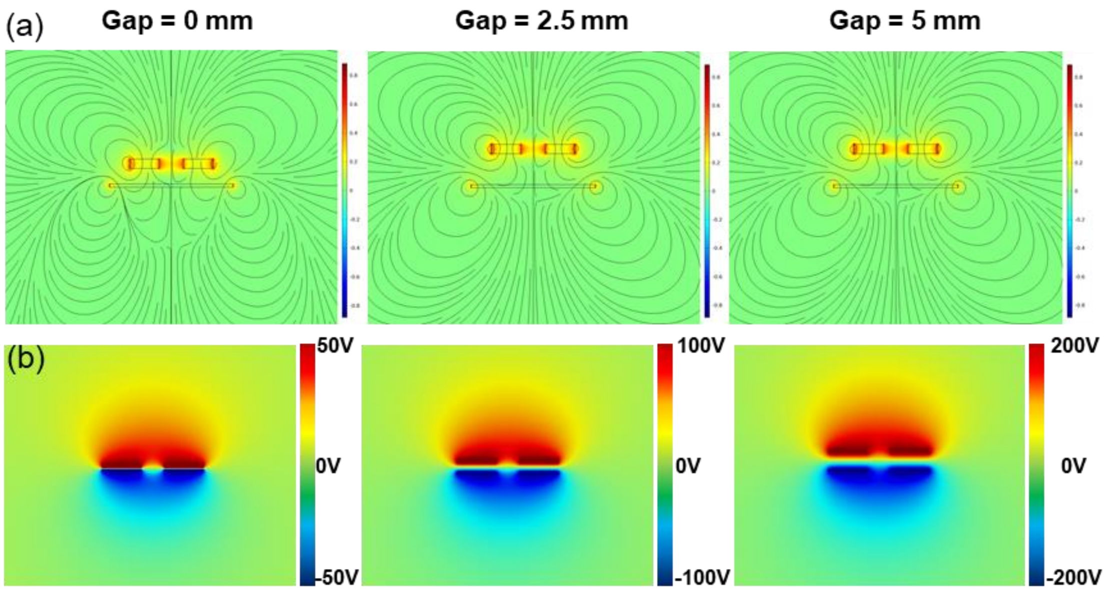

Figure S2. The COMSOL simulation of (a) the magnetic field distribution and (b) electric field distribution under different gaps between the tribo-charge materials.

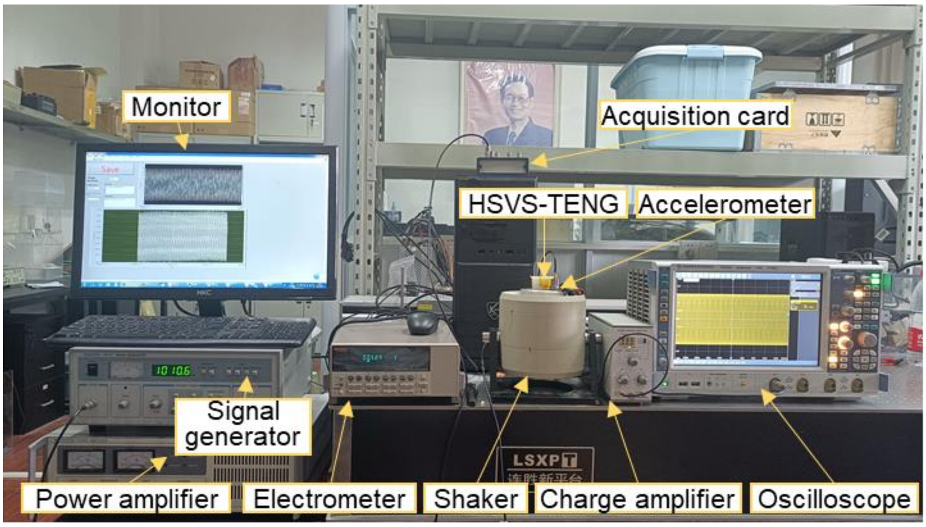

Figure S3. Experimental setup

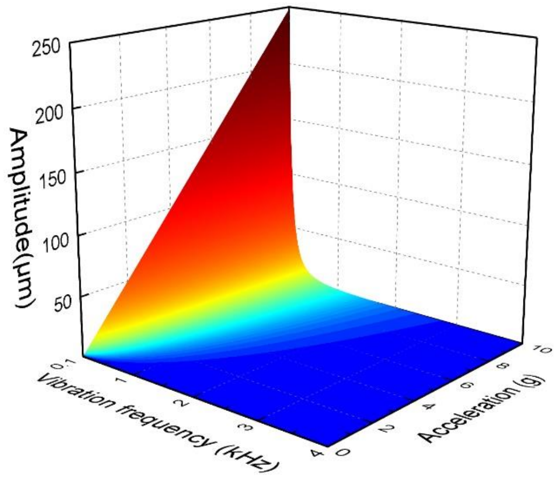

Figure S4. The relationship between the vibration frequency, acceleration, and amplitude.

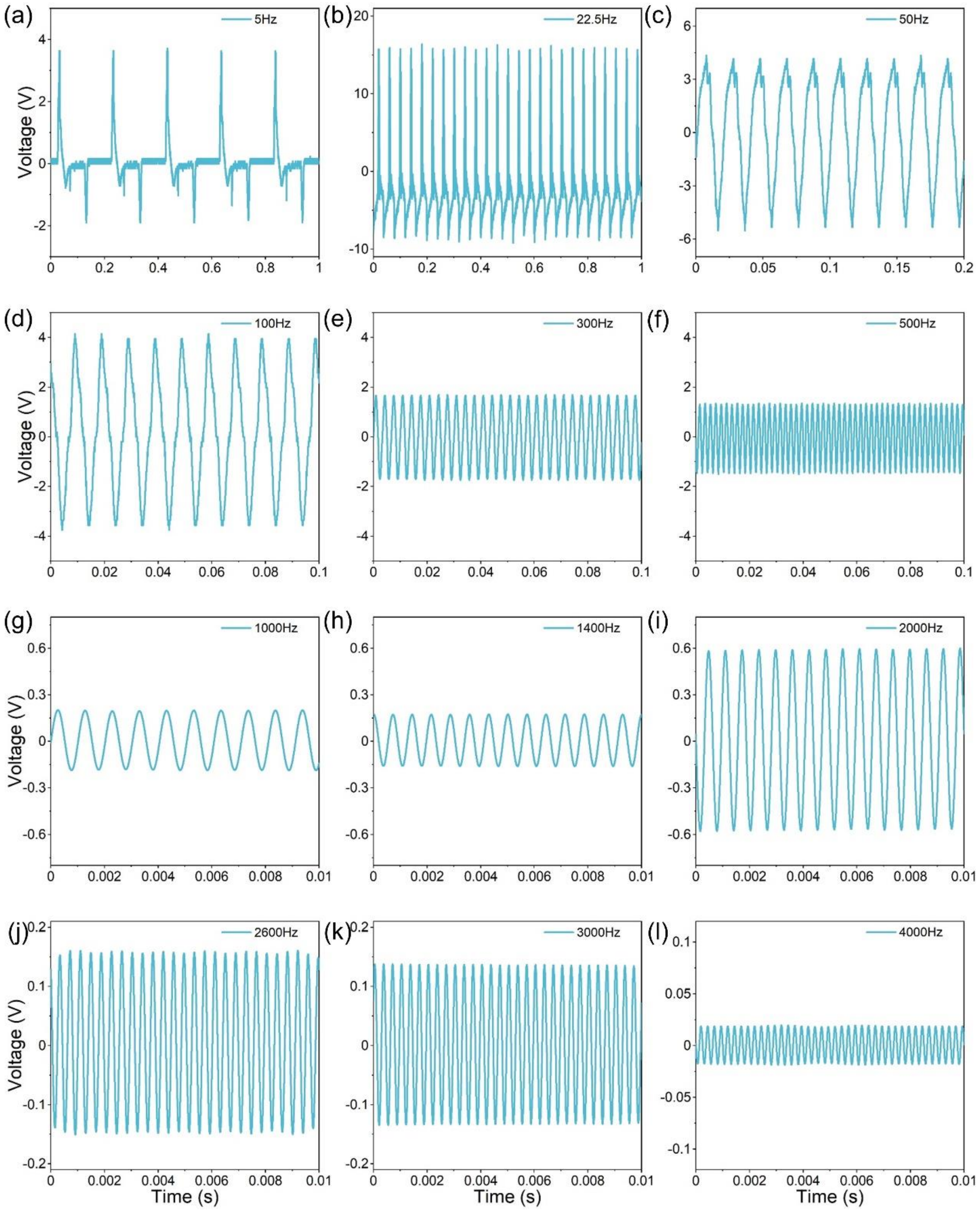

Figure S5. The open-circuit voltage of the HSVS-TENG under the vibration frequencies ranging from 5 - 4000 Hz and at an acceleration of 0.5 g.

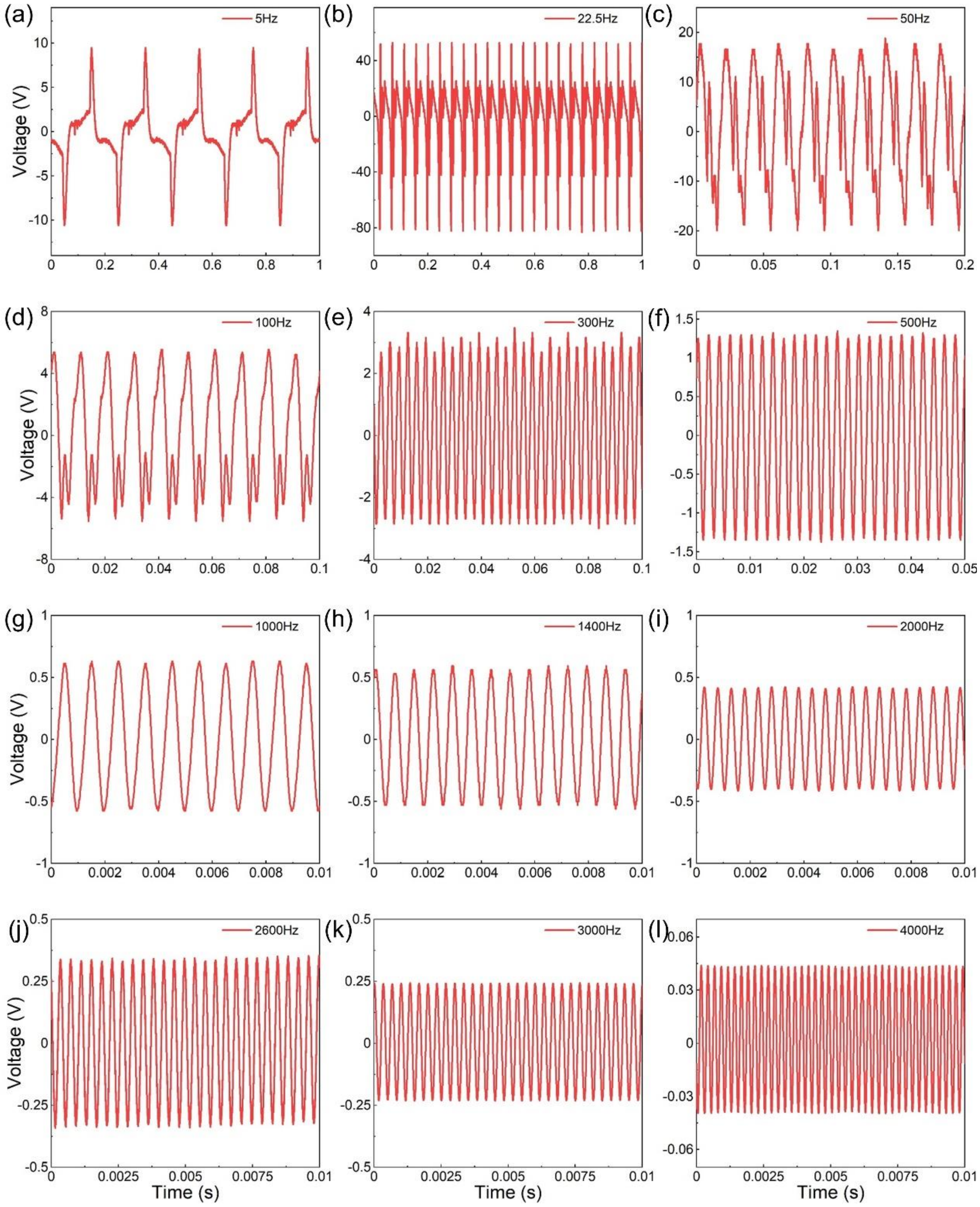

Figure S6. The open-circuit voltage of the HSVS-TENG under the vibration frequencies ranging from 5 - 4000 Hz and at an acceleration of 1 g.

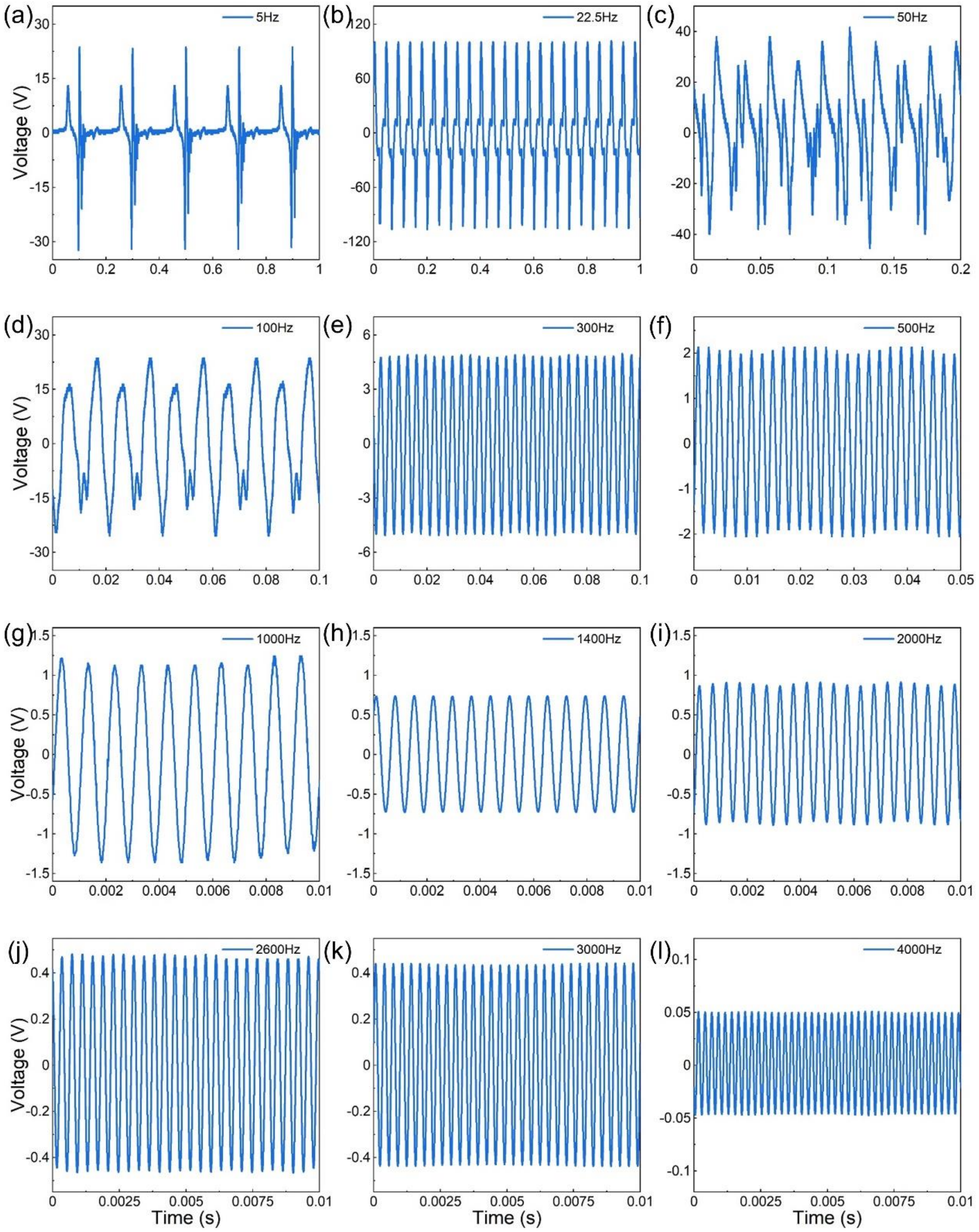

Figure S7. The open-circuit voltage of the HSVS-TENG under the vibration frequencies ranging from 5 - 4000 Hz and at an acceleration of 1.5 g.

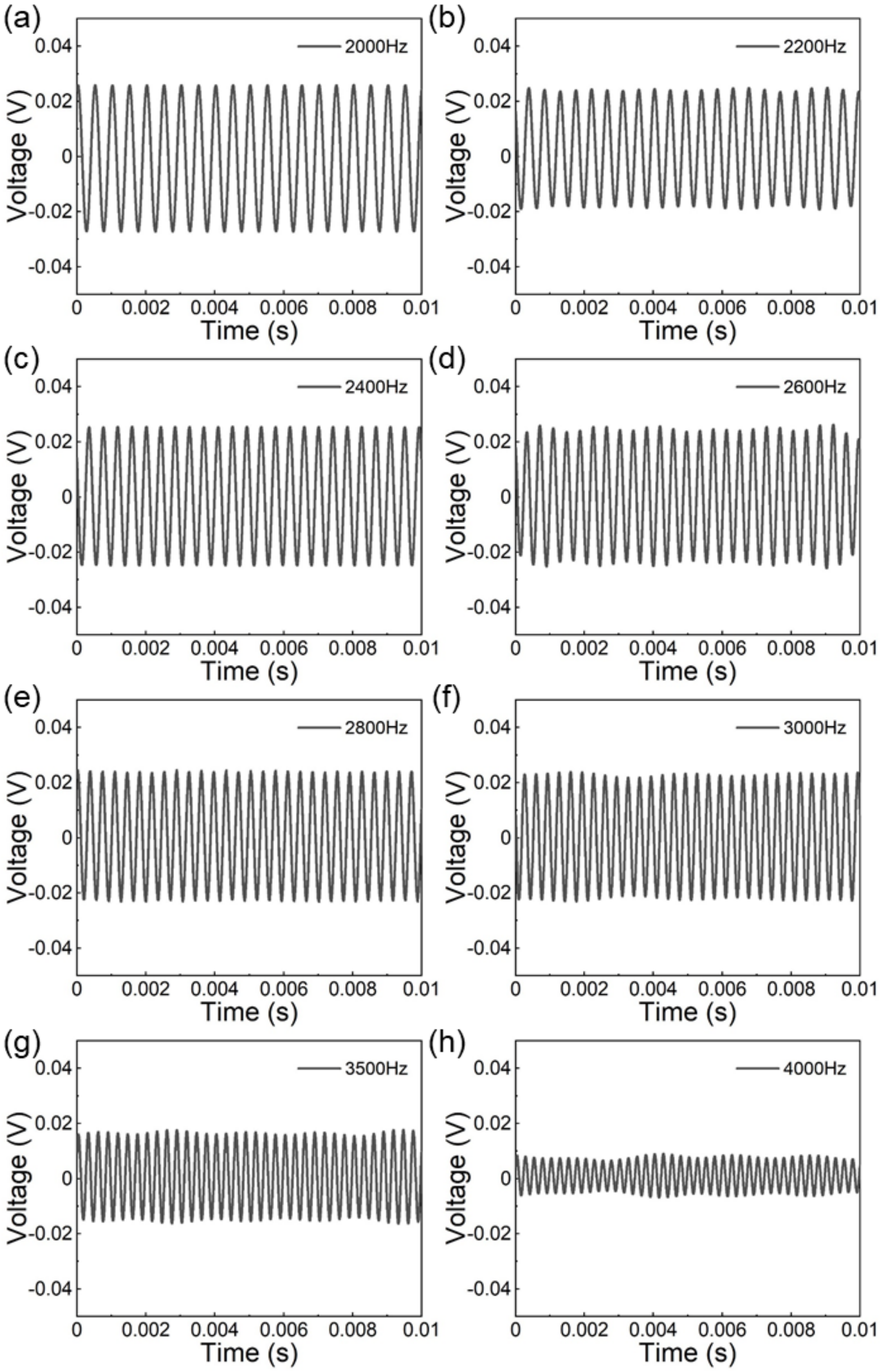

Figure S8. The open-circuit voltage of the HSVS-TENG under the vibration frequencies ranging from 2000 - 4000 Hz and at an acceleration of 0.1 g.

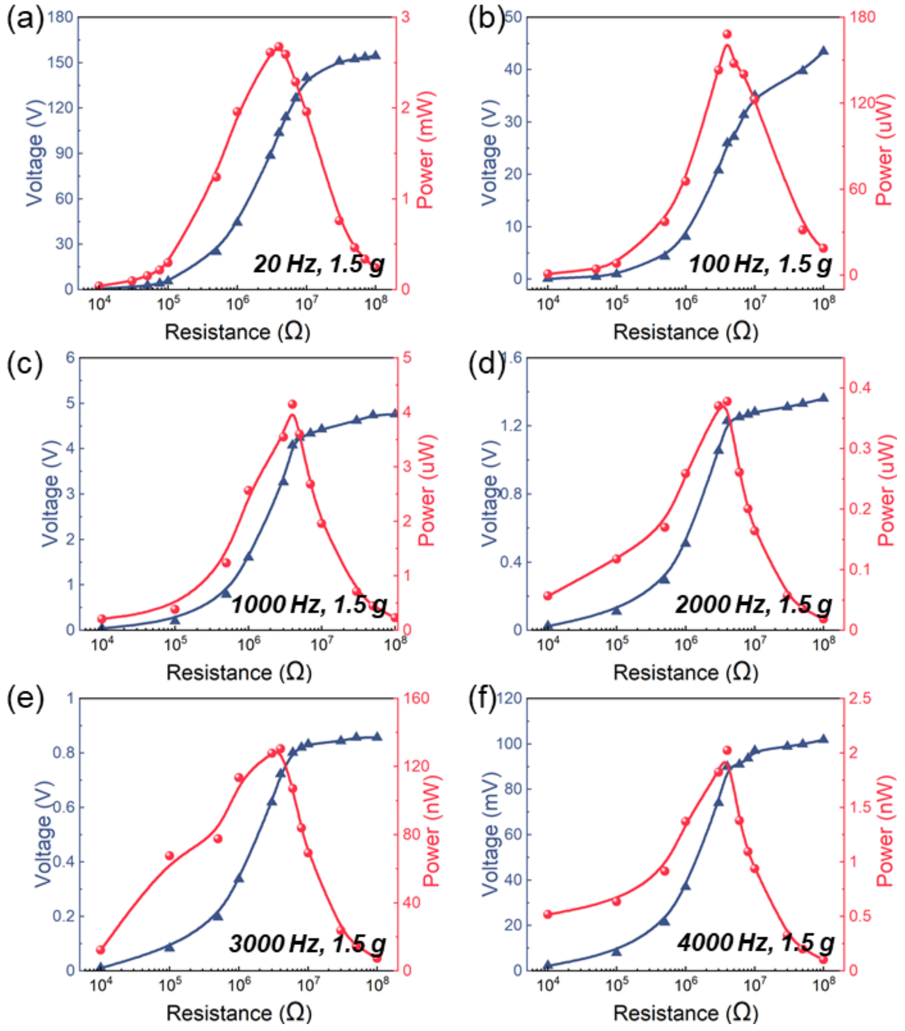

Figure S9. Peak power and voltage response of the TENG under various resistances at different vibration excitation.

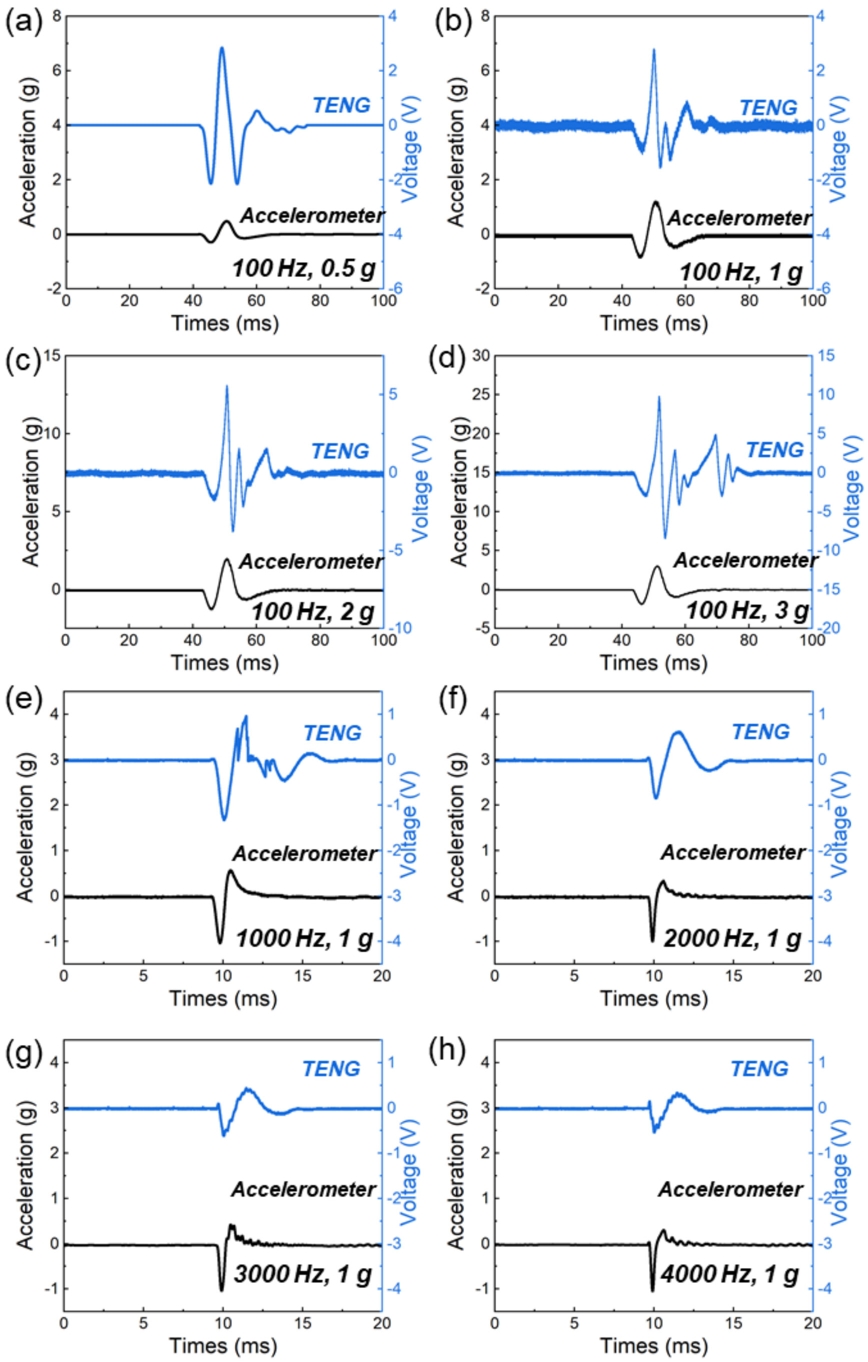

Figure S10. Corresponding voltage signals of the HSVS-TENG and accelerometer under different vibration pulse: a) 100 Hz, 0.5g, b) 100 Hz, 1g, c) 100 Hz, 2g, d) 100 Hz, 3g, e) 1000Hz, 1g, f) 2000Hz, 1g, g) 3000Hz, 1g, h) 4000Hz, 1g

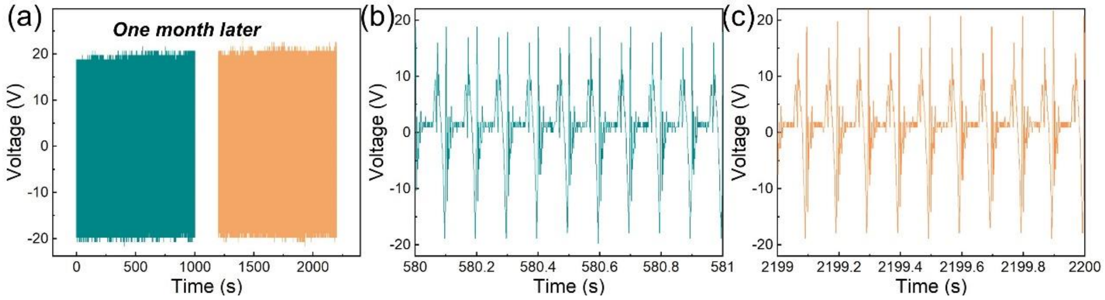

Figure S11. Long-term stability test of HSVS-TENG. a) Output stability of HSVS-TENG lased 24 000 working cycles under the vibration frequencies of 10 Hz and at an acceleration of 1 g. b) The output voltage signal from a month ago. c) The output voltage signal from a month later.

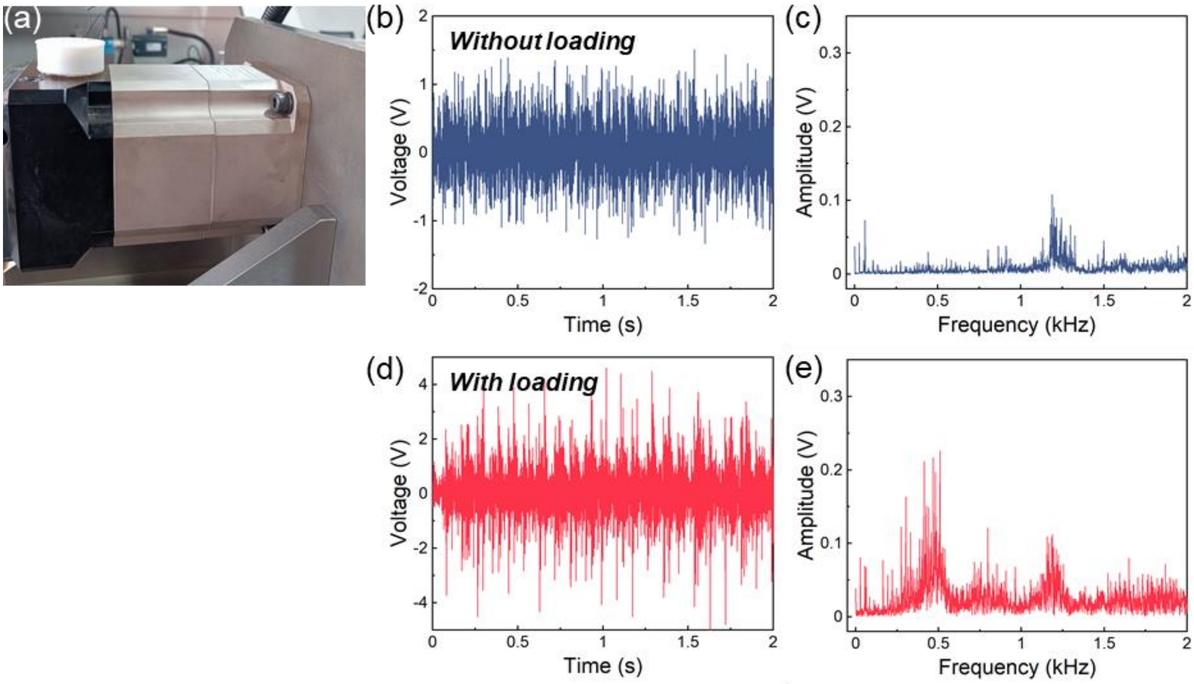

Figure S12. Output voltage signals by the HSVS-TENG at the different running states of the reducer a) photograph of the experiment. b) and c) time-domain and frequency without loading (R-N-L), d) and e) time-domain and frequency with loading (R-L).

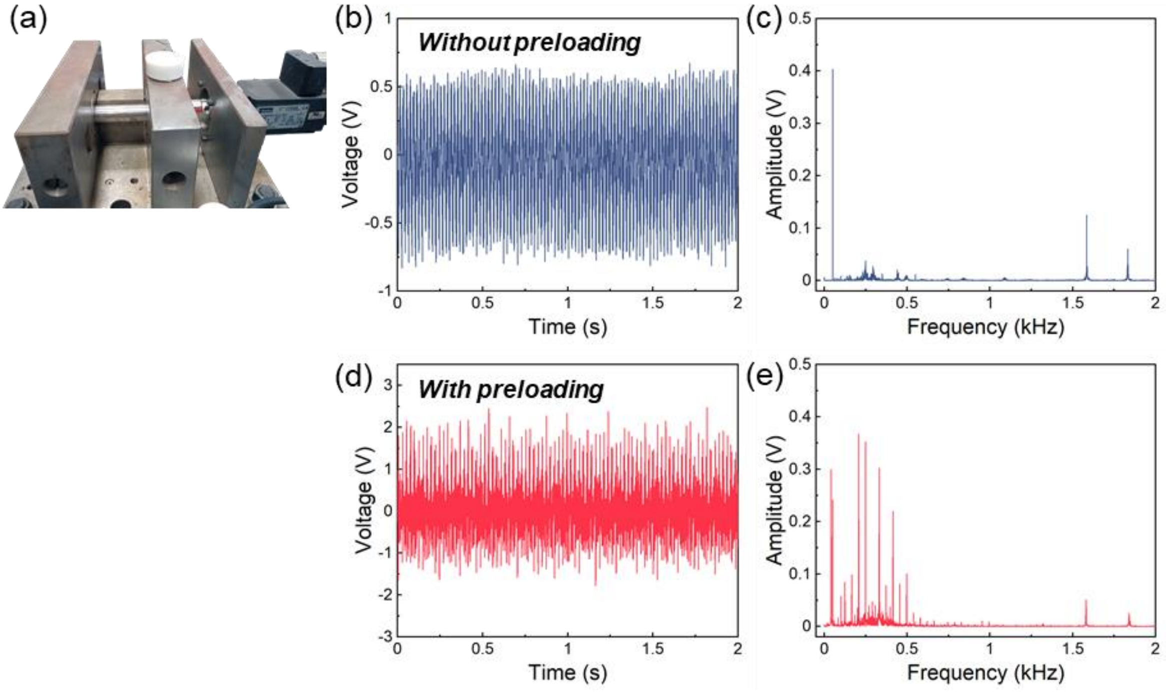

Figure S13. Output voltage signals by the HSVS-TENG at the different running states of the bearing a) photograph of the experiment. b) and c) time-domain and frequency without preloading (B-N-P), d) and e) time-domain and frequency with preloading (B-P).

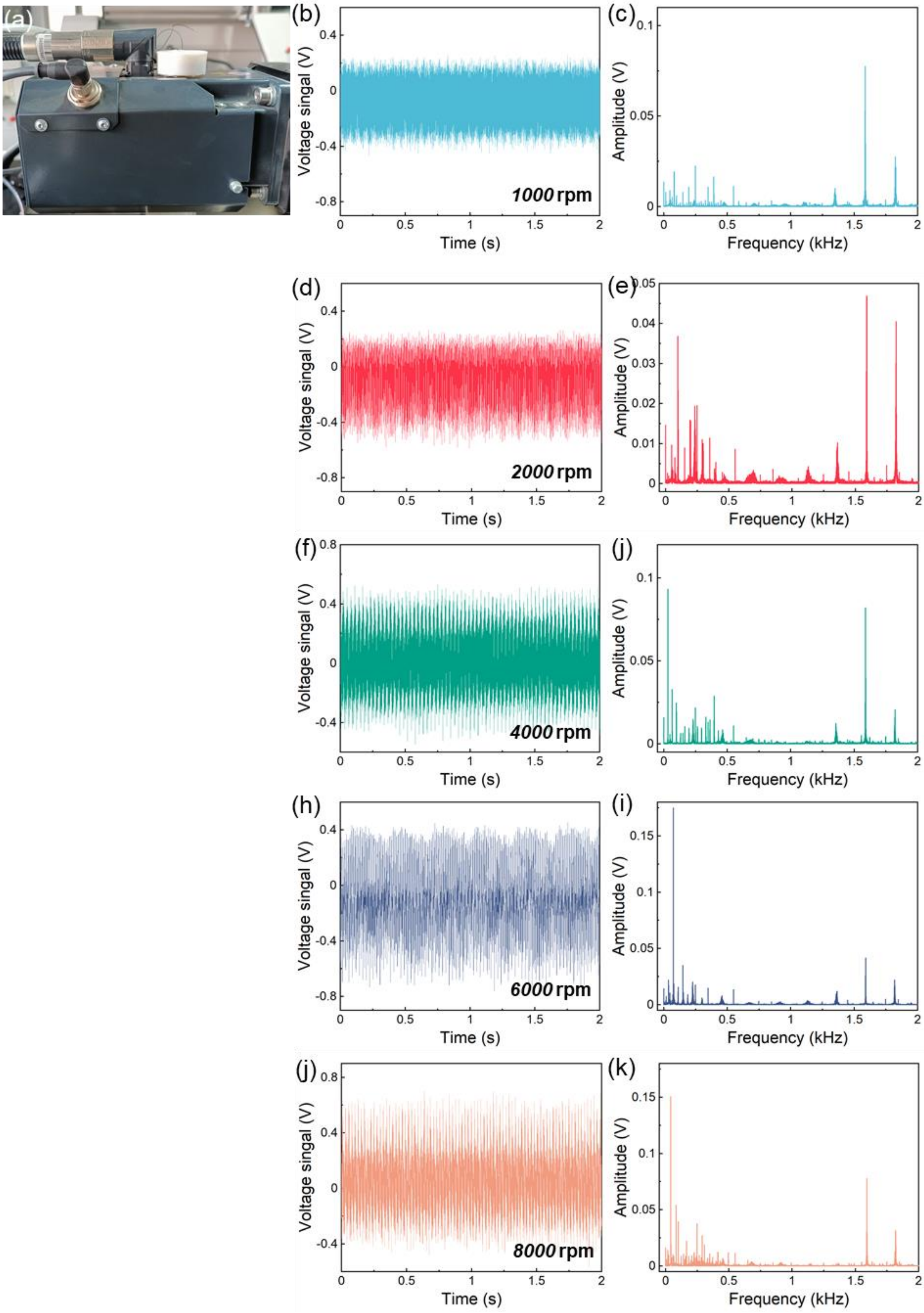

Figure S14. Output voltage signals by the HSVS-TENG at different rotational speeds of the motor. a) photograph of the experiment. b) and c) time-domain and frequency at 200 r/min(M-

200), d) and e) time-domain and frequency at 500 r/min (M-500), f) and g) time-domain and frequency at 1000 r/min (M-1k), h) and i) time-domain and frequency at 2000 r/min (M-2k), j) and k) time-domain and frequency at 2500 r/min (M-2.5k).

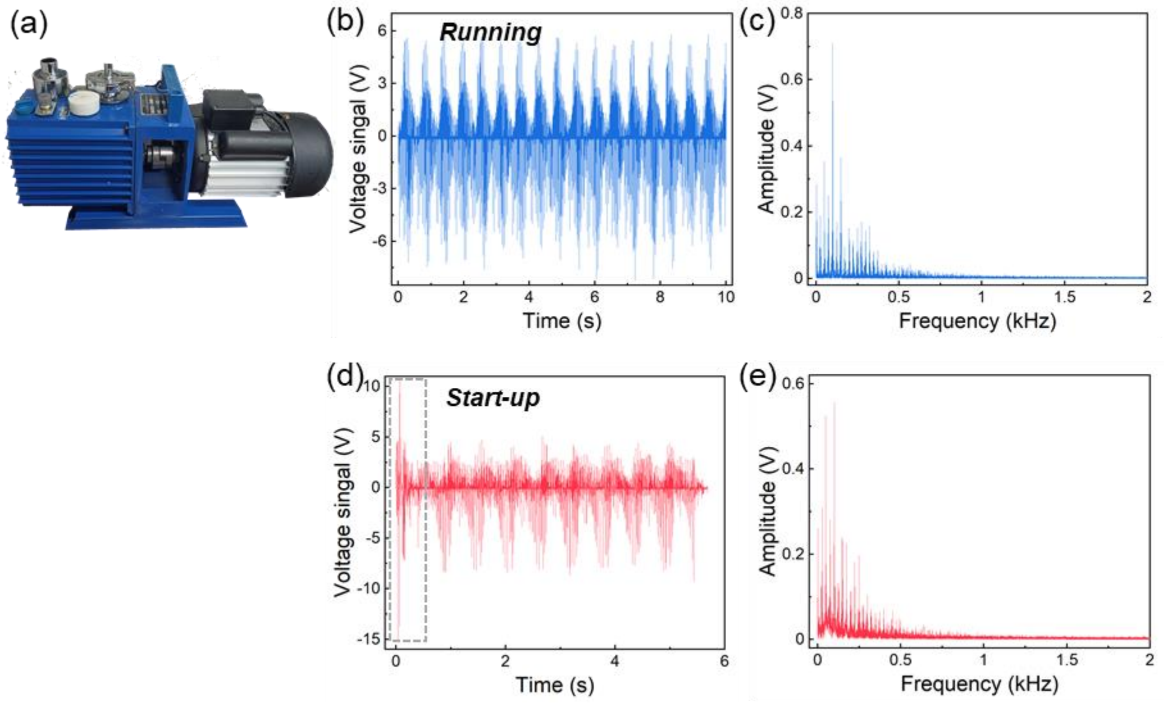

Figure S15. Output voltage signals by the HSVS-TENG at the different running states of the compressor. a) photograph of the experiment. b) and c) time-domain and frequency under normal running conditions (C-M), d) and e) time-domain and frequency under start-up conditions (C-SU).

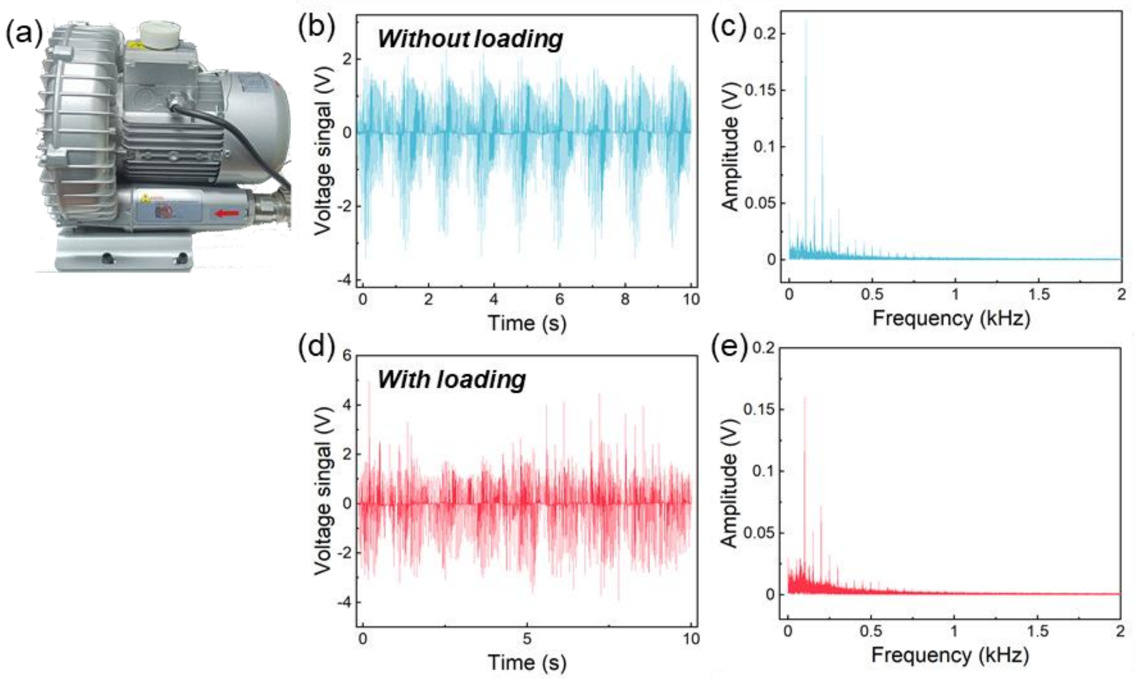

Figure S16. Output voltage signals by the HSVS-TENG at the different running states of the blast engineer. a) photograph of the experiment. b) and c) time-domain and frequency without loading (BE-N-L), d) and e) time-domain and frequency with loading (BE-L).

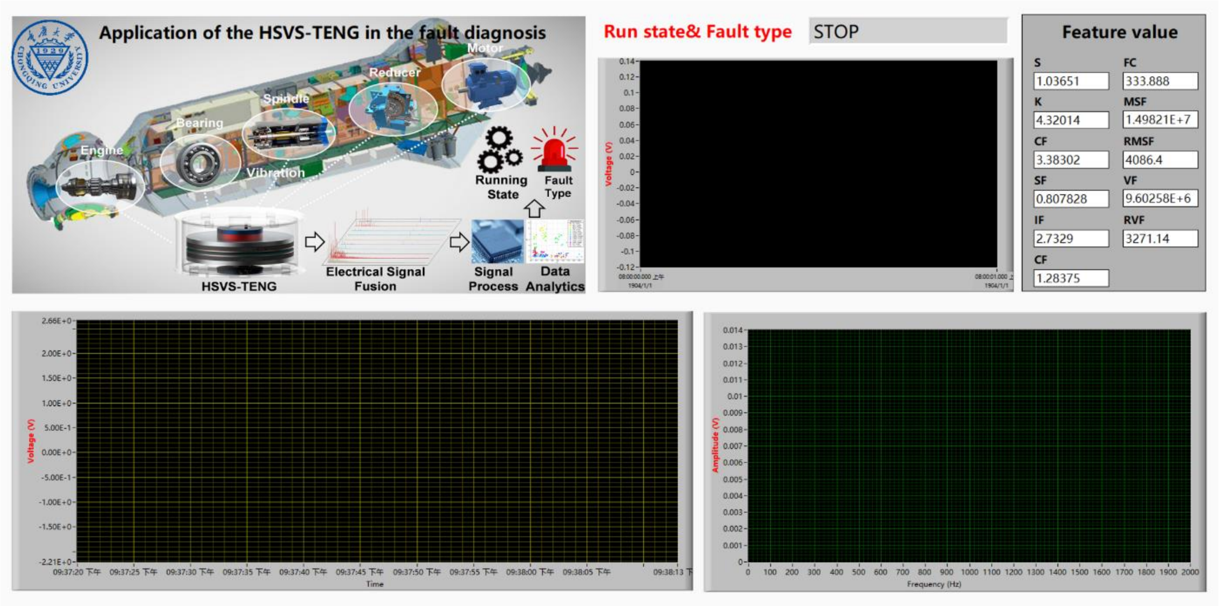

Figure S17. Software interfaces for intuitive demonstration.

Table S1. Comparison of self-powered vibration sensors in different studies.

**Time**	**Author**	**Technology**	**Frequency range (Hz)**	**Sensitivity (V/g)**	**Acceleration Range(g)**
2003	Manuel[1]	Piezoelectric	0.06~450	1	5
2013	Hu[2]	Capacitive	0~300	2	0~2
2014	Zhang[3]	Triboelectric	0~100	15.56	1~2
2016	Alessandro[4]	Capacitive	0~1500	0.12	0~3
2018	He[5]	Triboelectric	0~180	—	—
2018	Gao[6]	Inductive	0~500	5	
2019	Xiao[7]	Triboelectric	10-60	0.1~50	0-15
2019	Salauddin[8]	Triboelectric & Inductive	90	100	—
2020	Wang[9]	Triboelectric	5-60	10.78	0.1~0.6
2020	Li[10]	Inductive	0~3000	0.066	0.05-8.5
2022	Prutvi[11]	Triboelectric	0~400	0~14	—
2022	Mechamud[12]	Triboelectric	1-1200	44.56	0.281~6
2022	Zhao[13]	Triboelectric	10~2000	0.01~0.75	1~3.5
2022	Cheung[14]	Piezoelectric	0~90	1.152	0~1
Present	This work	Triboelectric	5~4000	0.32~134.9	0.1~6

Supplementary Note S1. The feature extraction of the SVM machine-learning algorithm

Most of the time-domain characteristic parameters of vibration signals are statistical characteristics, such as mean, effective value, variance, standard deviation, maximum value, minimum value, peak value, peak-to-peak value, etc[15]. These time-domain statistical characteristic indexes are characterized by dimension, and the eigenvalue size is strongly related to the working condition of the characterized object. Based on the dimensionless parameters of the time-domain statistical characteristics, time-domain dimensionless parameters such as skewness (*S*), kurtosis (*K*), crest factor (*CF*), shape factor (*SF*), impulse factor (*IF*), and clearance factor (*CLF*) can be as the feature of the time-domain voltage signal output by the HSVS-TENG[16]. the dimensionless parameters can be calculated as follow:

1) Skewness

$$S = \frac{\sum_{i=1}^{T}(x_i - \bar{x})^3}{(T-1)\sigma^3} \tag{N1}$$

2) Kurtosis

$$K = \frac{\sum_{i=1}^{T}(x_i - \bar{x})^4}{(T-1)\sigma^4} \tag{N2}$$

3) Crest Factor

$$CF = \frac{\max|x_i|}{\sqrt{\frac{1}{T}\sum_{i=1}^{T}x_i^2}} \tag{N3}$$

4) Shape Factor

$$SF = \frac{\sqrt{T\sum_{i=1}^{T}x_i^2}}{\sum_{i=1}^{T}|x_i|} \tag{N4}$$

5) Impulse Factor

$$IF = \frac{\max|x_i|}{\frac{1}{T}\sum_{i=1}^{T}|x_i|} \tag{N5}$$

6) Clearance Factor

$$CLF = \frac{\max|x_i|}{\left(\frac{1}{T}\sum_{i=1}^{T}\sqrt{|x_i|}\right)^2} \tag{N6}$$

where the x_i is the voltage signal value of the *i*th sampling point, $\bar{x}$ is the mean of the voltage

signal, the T is the number of sampling points, and the σ is the standard deviation of the voltage signal.

These dimensionless time-domain features have different meanings. Skewness and kurtosis characterize the severity of a fault by calculating the degree to which the signal deviates from a normal distribution. Crest factors can characterize fault development trends effectively. The shape factor can characterize the failure of key components. Impulse factor and clearance factor characterization of state change sensitively when faults occur, but poor stability.

The frequency-domain indexes of the vibration signals can characterize the fault state from another dimension. Typical frequency-domain characteristics of vibration signals are Frequency Centroid (*FC*), Mean Square Frequency (*MSF*), Root Mean Square Frequency (*RMSF*), Variance Frequency (*VF*), and Root Variance Frequency (*RVF*). It can be calculated as follow:

1) Frequency Centroid (*FC*)

$$FC = \frac{\int_0^\infty fA(f)df}{\int_0^\infty A(f)df} \tag{N7}$$

2) Mean Square Frequency (*MSF*)

$$MSF = \frac{\int_0^\infty f^2 A(f)df}{\int_0^\infty A(f)df} \tag{N8}$$

3) Root Mean Square Frequency (*RMSF*)

$$RSMF = \sqrt{\frac{\int_0^\infty f^2 A(f)df}{\int_0^\infty A(f)df}} \tag{N9}$$

4) Variance Frequency (*VF*)

$$VF = \frac{\int_0^\infty (f - FC)^2 A(f)df}{\int_0^\infty A(f)df} \tag{N10}$$

5) Root Variance Frequency (*RVF*)

$$RVF = \sqrt{\frac{\int_0^\infty (f - FC)^2 A(f)df}{\int_0^\infty A(f)df}} \tag{N11}$$

where the $A(f)$ is the amplitude of the frequency domain signal at frequency value f.

Supplementary Note S2. Machine-learning algorithms used in training and recognition

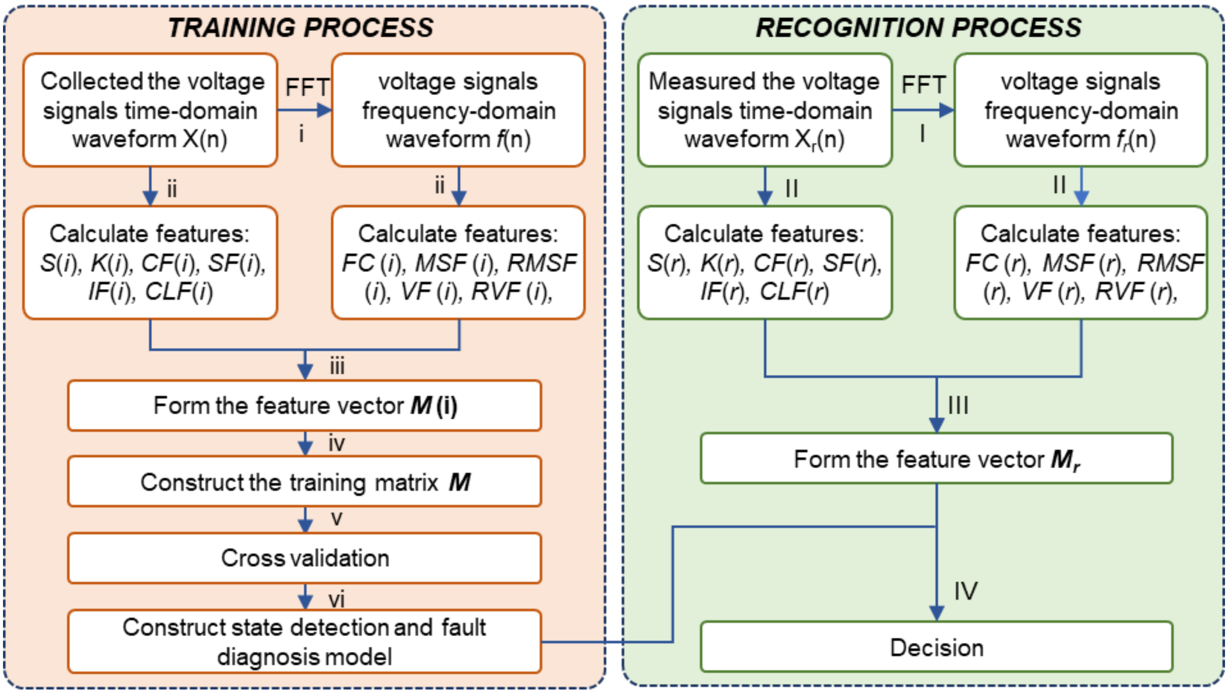

Figure S18 Training and recognition process of the machine-learning algorithm Cubic SVM

(1) Training process

i. The collected time-domain voltage signals of the HSVS-TENG X(n) were converted into frequency domain signals f(n) by fast Fourier transform (FFT), and the transformation process is as follows:

$$f(n) = \sum_{n=0}^{N-1} X(n) e^{-\frac{2\pi}{N}} \qquad \text{(N12)}$$

Where the X(n) is a discrete signal, N is the number of the signal.

ii. Calculating the features from the time-domain and frequency-domain signals, the process was show in **Supplementary Note S1.**

iii. the feature vector $\boldsymbol{M}(i)$ is

$$M(i) = [S(i) \quad K(i) \quad CF(i) \quad SF(i) \quad IF(i) \quad CLF(i) \quad FC(i) \quad MSF(i) \quad RMSF(i) \quad VF(i) \quad RVF(i)] \qquad \text{(N13)}$$

iv. the training matrix $\boldsymbol{M}$ can be written as:

$$M = \begin{bmatrix} S(1) & K(1) & CF(1) & SF(1) & IF(1) & CLF(1) & FC(1) & MSF(1) & RMSF(1) & VF(1) & RVF(1) & L(1) \\ & & \vdots & & & & \ddots & & & & & \vdots \\ S(i) & & & & & & \cdots & & & & & L(i) \end{bmatrix}$$

(N14)

where the L(i)=[L(1) L(2) ··· L(i)] is the label vector of the voltage signals generated by the vibration source.

v. The cross-validation is performed by importing $M(i)$ and $L(i)$ into the Cubic SVM software.

vi. Constructing the state detection and fault diagnosis model.

(2) Recognition process

I. The measured time-domain voltage signals of the HSVS-TENG X_r(n) were converted into frequency domain signals f_r(n) by fast Fourier transform (FFT), and the transformation process is as follows:

$$f_r(n) = \sum_{n=0}^{N-1} X_r(n) e^{-\frac{2\pi}{N}} \quad \text{(N15)}$$

Where the X_r(n) is a discrete signal, N is the number of the signal.

II. Calculating the features from the time-domain and frequency-domain signals, the process was show in **Supplementary Note S1.**

III. the feature vector $\boldsymbol{M}_\mathrm{r}$ is

$$M_\mathrm{r} = [S(r) \quad K(r) \quad CF(r) \quad SF(r) \quad IF(r) \quad CLF(r) \quad FC(r) \quad MSF(r) \quad RMSF(r) \quad VF(r) \quad RVF(r)] \quad \text{(N16)}$$

IV. Decision by importing $\boldsymbol{M}_\mathbf{r}$ and state detection and fault diagnosis model into the Cubic SVM software.

Reference

[1] M. Ruiz-Sandoval, B. F. Spencer, N. Kurata, presented at *4th International Workshop on Structural Health Monitoring, IWSHM*, **2003**.

[2] X. Hu, B. Wang, H. Ji, *COMPUT-AIDED CIV. INF.* **2013**, *28, 193*.

[3] H. Zhang, Y. Yang, Y. Su, J. Chen, K. Adams, S. Lee, C. Hu, Z. L. Wang, *Adv. Funct. Mater.* **2014**, *24, 1401*.

[4] A. Sabato, M. Q. Feng, Y. Fukuda, D. L. Carní, G. Fortino, *IEEE Sens. J.* **2016**, *16, 2942*.

[5] C. He, W. Zhu, G. Q. Gu, T. Jiang, L. Xu, B. D. Chen, C. B. Han, D. Li, Z. L. Wang, *Nano Res* **2018**, *11, 1157*.

[6] M. Gao, Y. Li, J. Lu, Y. Wang, P. Wang, L. Wang, *Int. J. Distrib. Sens. Netw.* **2018**, *14, 1550147718814469*.

[7] X. Xiao, X. Zhang, S. Wang, H. Ouyang, P. Chen, L. Song, H. Yuan, Y. Ji, P. Wang, Z. Li, M. Xu, Z. L. Wang, *Adv. Energy Mater.* **2019**, *9, 1902460*.

[8] M. Salauddin, R. M. Toyabur, P. Maharjan, M. S. Rasel, H. Cho, J. Y. Park, *Nano Energy* **2019**, *66*.

[9] Z. H. Wang, F. D. Zhang, N. Li, T. Yao, D. L. Lv, G. L. Cao, *Adv. Mater. Technol.* **2020**, *5*.

[10] Y. Li, Y. Wang, Q. Cao, J. Cao, D. Qiao, *IEEE Trans. Ind. Electron.* **2020**, *67, 560*.

[11] S. H. Prutvi, M. Korrapati, D. Gupta, *Meas. Sci. Technol.* **2022**, *33*.

[12] I. Mehamud, P. Marklund, M. Björling, Y. Shi, *Nano Energy* **2022**, *98, 107292*.

[13] H. Zhao, M. Shu, Z. Ai, Z. Lou, K. W. Sou, C. Lu, Y. Jin, Z. Wang, J. Wang, C. Wu, Y. Cao, X. Xu, W. Ding, *Adv. Energy Mater.* **2022**, *2201132*.

[14] Y. K. Cheung, Z. F. Zhao, H. Y. Yu, *Micromachines* **2022**, *13, 530*.

[15] Z. Yuan, S. Zhu, X. Yuan, W. Zhai, *Engineering Failure Analysis* **2021**, *119, 104906*.

[16] J. Yang, Y. Bai, J. Wang, Y. Zhao, *Meas. Sci. Technol.* **2019**, *30, 095009*.
